# Characteristics of different Mandarin pronunciation element perception: evidence based on a multifeature paradigm for recording MMN and P3a components of phonemic changes in speech sounds

**DOI:** 10.3389/fnins.2023.1277129

**Published:** 2024-01-08

**Authors:** Xiang Mao, Ziyue Zhang, Yijing Yang, Yu Chen, Yue Wang, Wei Wang

**Affiliations:** ^1^Department of Otorhinolaryngology Head and Neck Surgery, Tianjin First Central Hospital, Tianjin, China; ^2^Institute of Otolaryngology of Tianjin, Tianjin, China; ^3^Key Laboratory of Auditory Speech and Balance Medicine, Tianjin, China; ^4^Key Medical Discipline of Tianjin (Otolaryngology), Tianjin, China; ^5^Otolaryngology Clinical Quality Control Centre, Tianjin, China

**Keywords:** Mandarin Chinese speech perception, event-related potentials, Mandarin pronunciation multifeature paradigm, time domain analysis, source localization analysis

## Abstract

**Background:**

As a tonal language, Mandarin Chinese has the following pronunciation elements for each syllable: the vowel, consonant, tone, duration, and intensity. Revealing the characteristics of auditory-related cortical processing of these different pronunciation elements is interesting.

**Methods:**

A Mandarin pronunciation multifeature paradigm was designed, during which a standard stimulus and five different phonemic deviant stimuli were presented. The electroencephalogram (EEG) data were recorded with 256-electrode high-density EEG equipment. Time-domain and source localization analyses were conducted to demonstrate waveform characteristics and locate the sources of the cortical processing of mismatch negativity (MMN) and P3a components following different stimuli.

**Results:**

Vowel and consonant differences elicited distinct MMN and P3a components, but tone and duration differences did not. Intensity differences elicited distinct MMN components but not P3a components. For MMN and P3a components, the activated cortical areas were mainly in the frontal-temporal lobe. However, the regions and intensities of the cortical activation were significantly different among the components for the various deviant stimuli. The activated cortical areas of the MMN and P3a components elicited by vowels and consonants seemed to be larger and show more intense activation.

**Conclusion:**

The auditory processing centers use different auditory-related cognitive resources when processing different Mandarin pronunciation elements. Vowels and consonants carry more information for speech comprehension; moreover, more neurons in the cortex may be involved in the recognition and cognitive processing of these elements.

## 1 Introduction

Hearing perception refers to the process beginning with external sound stimulation of the auditory organ and ultimately producing emotional and cognitive responses. Furthermore, speech perception is a critical hearing-related cognitive process and an important skill involved in human communication with the outside world. As a tonal language, Mandarin Chinese has the following pronunciation elements for most syllables: the vowel, consonant, tone, duration, and intensity. The vowel and tone are required for each syllable in Mandarin; the consonant is also important component of speech comprehension; the duration and intensity are useful for perceiving the emotion of the speech. The accuracy of speech perception not only requires participation of the complete auditory pathway, consisting of the cochlea, auditory nerve, and nuclei in the brain stem and midbrain that transmit and encode auditory information, but also requires processing by higher level auditory-related cortical structures, namely, the temporal, parietal, and frontal lobes of the cerebral cortex (Patel and Iversen, 2007; Chandrasekaran and Kraus, 2010). Although previous research has indicated that these pronunciation elements are the structural components of syllables and are independent phonemic units (Gandour et al., 2003), in everyday communication, the auditory system is exposed to these pronunciation elements in combination, and it is rare to encounter each pronunciation element individually. However, these different pronunciation elements may have undergone different auditory-related cortical processing procedure. Therefore, it is meaningful to design a new method to separate these pronunciation elements from the syllables to analyze them independently.

Electroencephalogram (EEG) is a brain imaging technique. The most prominent feature of EEG is its ultrahigh temporal resolution, which is particularly advantageous for studying the rapid dynamic changes in functional brain networks during higher-order cognitive processes, which often last for tens of milliseconds (Müller-Putz, 2020). Moreover, the use of high-density EEG (up to 256 electrodes) could overcome the poor spatial resolution problem in locating the source of cortical processing (Lantz et al., 2003; Väisänen and Malmivuo, 2008). Event-related potentials (ERPs), which are derived from EEG data, can reflect auditory-related cortical processing (Luo et al., 2006). ERPs provide temporally specific information on the various stages of auditory processing and contain several stereotyped neural components associated with perception and behavior. Among the ERP components, the mismatch negativity (MMN) and P3a are widely used in hearing-related research (Liang et al., 2014; Gaebler et al., 2015). The MMN and P3a are elicited by rare deviant stimuli in series of standard stimuli; subjects do not need to perform a specific task or focus their attention on the stimuli. The MMN appears to be generated in the frontotemporal cortex from 155 to 225 ms after stimulus onset (Deouell, 2007; Pulvermüller and Fadiga, 2010; Halgren et al., 2011). It reflects the preattentive detection of deviant events and serves as an indicator of the accuracy of neural auditory discrimination (Näätänen et al., 2007). Numerous studies have demonstrated that a greater amplitude and shorter latency of MMN could indicate better auditory neural development, and vice versa (Fu et al., 2016; Näätänen et al., 2017; Ni et al., 2021). The P3a appears to be distributed across frontal, parietal and temporal cortical regions following the MMN (Takahashi et al., 2013). Compared with the MMN, the P3a is an indicator of the sensitivity of involuntary attention allocation, possibly reflecting higher-level process-detecting events that may require further processing (Escera and Corral, 2007; Nager et al., 2007). Similar to the MMN, a larger and earlier P3a to subtle deviants is associated with highly accurate auditory discrimination, indicating a better capability to detect and interpret the auditory information input (Torppa et al., 2012; Putkinen et al., 2013a,b). Therefore, MMN and P3a were suitable neuroelectrophysiological markers to reveal the central auditory processing of phonemic deviant stimuli.

Most hearing-related studies have used the oddball stimulation paradigm to evoke MMN and P3a components (Ni et al., 2021). The oddball paradigm has only one deviant stimulus in each auditory sequence. The standard stimulus is a high probability event, accounting for 85% of the auditory sequence, and the deviant stimulus is a low-probability event, accounting for 15%. This traditional paradigm can only be used to analyze and evaluate one deviant stimulus at a time; if researchers want to evaluate multiple deviant stimuli, they need to repeat the test for each deviant stimulus, which will inevitably cause fatigue and boredom in the subjects; moreover, the state of each test could not be made completely consistent. To optimize the measurement of the MMN components to different kinds of phonemic features, Näätänen et al. (2004) presented five types of phonemic changes in one sequence of auditory stimuli. This multifeature paradigm integrates multiple deviant stimuli into one paradigm while ensuring that the number of deviant stimuli meets the requirements for superposition averaging (Putkinen et al., 2012). Because each deviant stimulus only involves a deviation in one of the five sound features of the standard stimulus (for example, with ba-pa, only the consonant is changed, while the vowel, tone, intensity, and duration are unchanged), the ERP evoked by the multifeature paradigm is comparable to that of the traditional oddball paradigm and significantly reduces testing time. Additionally, the reliability of the multifeature paradigm has been verified in several studies (Grimm et al., 2008; Pakarinen et al., 2009; Niemitalo-Haapola et al., 2013).

To fill the knowledge gaps regarding the characteristics of auditory-related cortical processing of different Mandarin pronunciation elements, an EEG study was conducted in normal-hearing Mandarin Chinese speakers. First, we designed and constructed a Mandarin pronunciation multifeature paradigm consisting of a standard stimulus and five different phonemic deviant stimuli. Second, the EEG data were recorded with 256-electrode high-density EEG equipment. Third, we conducted time-domain analysis and source localization analysis to demonstrate the waveform characteristics of the MMN and P3a components and locate the sources of the cortical processing underlying the MMN and P3a components following different stimuli. This study facilitates the deeper understanding of the characteristics of the auditory cortical processing procedure in response to phonemic changes in speech sounds.

## 2 Materials and methods

### 2.1 Study participants

From August 2022 to October 2022, 22 young staff members and graduate students (18 males, 4 females) between the ages of 22 and 34 years (median: 30, IQR: [23, 32]) volunteered to participate in this study. None reported a history of drug use or substance abuse, mental or neurological diseases, head trauma, or hearing impairment. Moreover, an audiologist checked the ear canals all the participants for cerumen and foreign matter using an otoscope. Pure tone hearing thresholds from 250 to 8,000 Hz were measured using an audiometer (Grason-Stadler, GSI). All hearing thresholds were below 20 dB HL (mean: 13.07, SD: 2.86). All the participants were right-handed.

### 2.2 Ethics statement

In compliance with Declaration of Helsinki, the Medical Ethics Committee of Tianjin First Central Hospital approved the research protocol. The review number is 2020N114KY.

### 2.3 Test procedure and Mandarin pronunciation multifeatureparadigm design

The test was conducted in a soundproof room with a background noise level of less than 30 dB (A). The intersection point of the connecting line between the center of both ears and the midline of the room was used as the reference test point. The center point of the connecting line between the two loudspeakers was 1 m from the reference test point with a 45° angle of incidence and the same height. Participants watched a silent, subtitled video during the experiment and were asked to minimize movement and eye-blinking.

**FIGURE 1:**
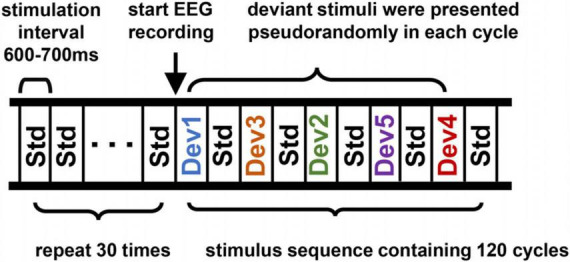
Schematic of the Mandarin phonemic multifeature paradigm. Std, standard stimulus; Dev 1, tone deviation; Dev 2, duration deviation; Dev 3, vowel deviation; Dev 4, consonant deviation; Dev 5, intensity deviation.

The Mandarin pronunciation multifeature paradigm consisted of a standard stimulus (50%) and five different types of deviants (10% each), as shown in Figure 1. The standard stimulus was a 70 dB SPL syllable/bā/that was 200 ms in duration with a 50 ms rise and fall time. The deviants were as follows: deviant 1 was a tone change from/bā/to/bà/; deviant 2 was a duration change from 200 to 300 ms; deviant 3 was a vowel change was from/bā/to/bī/; deviant 4 was a consonant change from/bā/to/pā/; and deviant 5 was an intensity change from 70 to 77 dB. The Mandarin syllables were recorded by professional male announcers in an acoustically shielded room and were normalized using Cool Edit Pro software (Syntrillium Software Corporation).

At the beginning of the stimulus sequence, 30 standard stimuli were presented to form a memory trace but these epochs were not included in subsequent stacking averages. Afterward, the standard and deviant stimuli were presented in a cycle consisting of five standard stimuli and five different deviant stimuli, with the whole stimulus sequence containing 120 cycles. The five deviant stimuli were presented pseudorandomly in each cycle, so it was never the same deviant that preceded or followed a standard stimulus. Each stimulation interval was drawn from a 600 to 700 ms random distribution to avoid expectation effects. The whole stimulus sequence included the 600 standard stimulus presentations and 120 presentations of each deviation stimulus and lasted approximately 20 min in total. The timing and presentation of all stimuli were controlled by a computer running E-Prime 3.0 (Psychology Software Tools Corporation, USA) software.

### 2.4 EEG recording

Electroencephalogram was recorded with the EGI GES400 (EGI Corporation, USA) using a GSN-HydroCel™-257 saline electrode cap, which was soaked in potassium chloride solution for 10 min before the test to enhance conductivity. During the data collection process, the electrode impedance was controlled within 50 KΩ. During the experiment, Net Station Acquisition 5.4.3-R software was used to record the EEG data, with a sampling rate of 1 kHz and Cz being considered as the reference electrode.

### 2.5 EEG data preprocessing

The raw EEG data were preprocessed using the EEGLAB open-source toolbox for MATLAB (R2021a) software (MathWorks Inc., USA). The data preprocessing steps included the following: (1) high-pass filtering at 1 Hz, low-pass filtering at 30 Hz, and notch-filtering at 50 Hz to reduce electrode drift, electromyography (EMG) signals, and power line interference, respectively; (2) downsampling to 250 Hz; (3) interpolation of electrodes with poor signal quality and manual removal of epochs with poor signal quality; (4) referencing to a full head average reference (Hu et al., 2018); (5) epoching of EEG data from 150 ms before to 750 ms after the stimulus onset and baseline correction using the 150 ms period before the stimulus onset; (6) use of independent component analysis (ICA) to remove artifacts such as blinking, glancing, and EMG signals from skeletal muscle or the myocardium (Chaumon et al., 2015); (7) and removal of any remaining artifacts greater than 100 μV.

### 2.6 EEG data time-domain analysis

The MMN and P3a components were defined as the waveforms of the deviant stimuli minus those of the standard stimulus that were averaged across the nine electrodes surrounding the Fz and Cz electrode (Friedman et al., 2001; Näätänen et al., 2017). Time windows were determined separately for each deviant stimulus based on the MMN and P3a waveforms by determining the latency at the lowest and highest value of the MMN and P3a waveforms in the 150–250 and 200–300 ms time windows after stimulus presentation (Torppa et al., 2012; Putkinen et al., 2013b). The mean amplitude topography was calculated using the time window of ±20 ms around the latency of the lowest and highest value time point.

### 2.7 Statistical analysis of EEG time domain data

The latencies and amplitudes followed normal distributions, so repeated measures of one-way analysis of variance (RM-ANOVA) was used to compare the differences in the latencies and amplitudes of MMN and P3a components among the five deviant stimuli. Before the RM-ANOVA, Mauchly’s test was conducted to test the assumption of sphericity. If Mauchly’s test indicated that the assumption of sphericity had been violated, the degrees of freedom were corrected using Greenhouse–Geisser estimates of sphericity (Greenhouse and Geisser, 1959). Bonferroni was used to *post hoc* analyses. IBM SPSS Statistics 20.0 (IBM Inc.) software was used for the statistical analysis, and *p* < 0.05 was considered to indicate a significant difference. GraphPad Prism 5 (Graph Pad Software Inc.) was used to construct scatterplots.

### 2.8 Source localization analysis

Source localization was implemented using the FieldTrip (version 20220819) toolbox for MATLAB (R2021a). For source localization, the EEG data from all electrodes were rereferenced to the average of all electrodes (common average reference). EGI GSN-HydroCel™-257 Sensor Net electrode locations were used in the source reconstruction for all subjects, and the electrodes were aligned to a volume conduction model. The volume conduction model was calculated using the boundary element method (Hallez et al., 2007) using a standard brain of a male subject’s T1 images acquire with MRI equipment (Siemens 3.0T MAGNETOM Trio Tim MRI equipment) at the Department of Radiology, Tianjin First Central Hospital. This standard volume conduction model was used for all subjects. Minimum norm estimates (MNEs) were used to solve the inverse problem of EEG source localization. MNE is based on a search for a solution with minimum power and corresponds to Tikhonov regularization (Grech et al., 2008). This estimate is suitable for distributed source models where the dipole activity is likely to extend over some areas of the cortical surface. The location of the MMN and P3a component sources was defined as areas with 10 × log_10_ (deviant stimulus power / standard stimulus power) values in the top 40% (McMackin et al., 2019). The mean sources of the MMN and P3a components were calculated using the ±20 ms time window around the identified latency of the trough or peak for each deviant stimulus.

The differences in the sources of the MMN and P3a components evoked by different deviant stimuli were tested for statistical significance using a cluster-based random permutation procedure (a non-parametric statistical test) to identify consistent differences in voxel clusters (Maris and Oostenveld, 2007; Moreno et al., 2015). This method could control the familywise error rate caused by the many statistical comparisons at the critical alpha level (Bullmore et al., 1999). First, RM-ANOVA was computed, and the *p*-values were thresholded (α = 5%) to determine the difference between stimuli for voxels during the identified MMN and P3a component time windows. Second, all voxels whose *F*-value is larger than the set threshold were selected. Third, significant voxels were clustered based on spatial adjacency, and a cluster-level test statistic was calculated by computing the sum of all *F* values in the cluster to assess the statistical significance of each cluster. Fourth, the significance of each cluster-level statistic was estimated by comparing the cluster statistic to a permutation distribution derived from the data, with cluster statistics falling in the highest 5th percentile considered significant. The permutation distribution is the distribution of the test statistic under the null hypothesis that the distributions of the five stimuli are identical. The permutation distribution was obtained by randomly permuting the data 5,000 times.

## 3 Results

### 3.1 Time domain characteristics of MMN/P3a components

**FIGURE 2:**
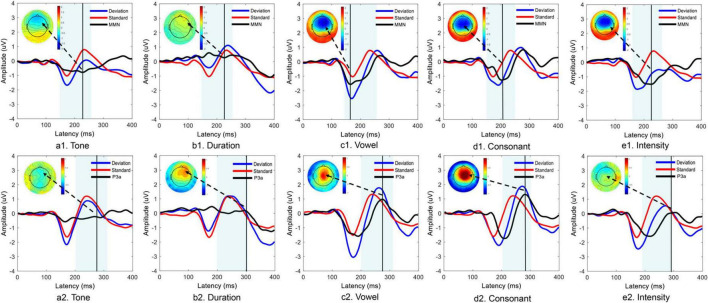
Time domain analysis of MMN and P3a components elicited by five different deviant stimuli. Panels **(a1–e1)** show the MMN component data, and panels **(a2–e2)** show the P3a component data.

For the MMN components, the features of the waveforms elicited by each deviant stimulus were different. The vowel, consonant and intensity deviants elicited distinct MMN component waveforms, and obvious negative areas were also found in the frontal region in the topographic map. However, no distinct MMN components were observed with the tone and duration deviant stimuli. For the P3a components, the features of the waveforms elicited by each deviant stimulus were also different. The vowel and consonant deviants elicited distinct P3a components. The tone and duration also did not elicit distinct P3a components. Notably, the intensity deviants generally elicited distinct MMN components but did not elicit distinct P3a components (Figure 2).

### 3.2 Comparison of the characteristics of MMN and P3a components for different deviants

The RM-ANOVA showed that there were statistically significant main effect of the stimulus type on the amplitude of the MMN [*F*(4,76) = 30.099, *p* < 0.001, $\eta_{p}^{2}=0.613$], the latency of the P3a [*F*(2.7,51.6) = 7.661, *p* < 0.001, $\eta_{p}^{2}=0.287$] and the amplitude of the P3a [*F*(4,76) = 20.568, *p* < 0.001, $\eta_{p}^{2}=0.520$]. However, the main effect of the stimulus type on the latency of the MMN [*F*(2.5,46.7) = 2.131, *p* = 0.120, $\eta_{p}^{2}=0.101$] was not statistically significant (Table 1).

**TABLE 1:** Comparison of the characteristics of the MMN and P3a components between different deviants.

	*X*	*S*	*F*	*P*	$\eta_{p}^{2}$
**MMN**					
**Latency (s)^a^**					
Tone	0.205	0.035			
Duration	0.200	0.040			
Vowel	0.189	0.027	2.131	0.120	0.101
Consonant	0.198	0.026			
Intensity	0.216	0.023			
**Amplitude (μ V)**					
Tone	-1.269	0.548			
Duration	-0.134	0.441			
Vowel	-1.972	0.984	30.099	<0.001	0.613
Consonant	-1.783	0.852			
Intensity	-1.882	0.927			
**P3a**					
**Latency (s)^a^**					
Tone	0.258	0.031			
Duration	0.254	0.032			
Vowel	0.274	0.013	7.661	<0.001	0.287
Consonant	0.279	0.011			
Intensity	0.281	0.016			
**Amplitude (μ V)**					
Tone	0.288	0.525			
Duration	0.667	0.478			
Vowel	0.994	0.689	20.568	<0.001	0.520
Consonant	1.512	0.838			
Intensity	0.273	0.765			

^a^Mauchly’s test indicated that the assumption of sphericity had been violated in the MMN and P3a latency data (latency_MMN_: χ^2^ = 26.009, P = 0.002; latency_P3a_: χ^2^ = 20.298, P = 0.017). Therefore, their degrees of freedom were corrected using Greenhouse–Geisser estimates of sphericity (latency_MMN_: ε = 0.615; latency_P3a_: ε = 0.679).

**FIGURE 3:**
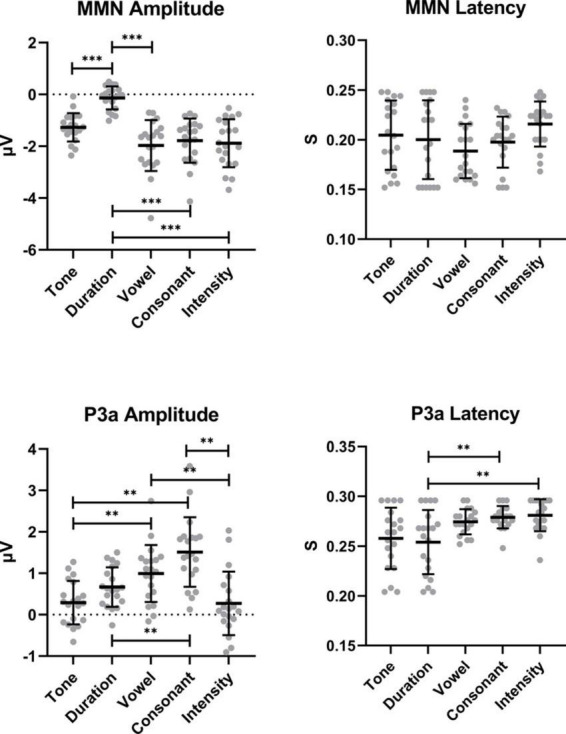
*Post hoc* analyses after RM-ANOVA. *Post hoc* analyses using the Bonferroni correction. **0.001 < *P* < 0.01, ***P < 0.001. The 0.01 < *P* < 0.05 marker is not shown to simplify the figure.

With regard to the MMN amplitudes, the duration deviant elicited a significantly lower amplitude than that of all the other deviant stimuli; this difference contributed the most to the overall between-group differences. The MMN amplitudes for the vowel, consonant and intensity deviants were very similar. With regards to the P3a amplitude, there appeared to be some differences among the groups; notably, the P3a amplitude for the consonant deviant was significantly higher than that of the other deviant stimuli, except the vowel deviant. Unlike the MMN amplitude, the P3a amplitude for the intensity deviant is almost zero. In terms of the P3a latency, the between-group difference is mainly focused on the duration deviant (Figure 3).

### 3.3 Source localization of MMN/P3a components

**FIGURE 4:**
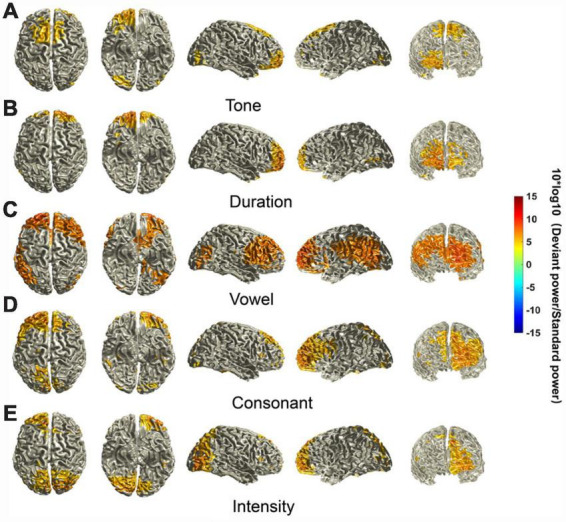
Source localization of MMN components. The location of MMN sources was defined as those with a 10 × log10 (deviant stimuli power / standard stimuli power) value in the top 40%. The mean sources of the MMN components were calculated using the ±20 ms time window around the identified time point of the trough for each of the different deviants.

We identified some characteristics of the MMN-activated cortical areas. First, activated cortical areas for the different deviant stimuli were all mainly in the frontal lobe, including the posterior lateral prefrontal cortex, frontopolar region and frontal-orbital area (Brodmann areas 9, 10, and 11). However, there was laterality in the dominant regions, i.e., the sources elicited by the tone and duration deviants were biased to the right, and the sources elicited by the vowel, consonant and intensity deviants were biased to the left. Second, the intensities of the neural activity for different deviant stimuli were different. The sources elicited by the vowel deviant seemed to have the highest intensity of neural activity. However, there was little difference in the intensity of the sources elicited by the other deviants. Third, the regions of the activated cortex elicited by the five deviant stimuli were also different. The sources elicited by the vowel had additional areas of strong activation in the pars opercularis, pars triangularis and superior lateral frontal cortex (Brodmann areas 44, 45, and 46) in the right hemisphere and angular convolution and supramarginal gyrus (Brodmann areas 39 and 40) in the left hemisphere. The sources elicited by the consonant deviant also had large areas of activation, including the superior lateral frontal cortex (Brodmann area 46) and part of the pars opercularis and pars triangularis (Brodmann areas 44 and 45). The sources elicited by the tone and duration deviants have relatively small areas of activation. The sources elicited by the intensity deviant had additional areas of activation in the primary visual cortex, secondary visual cortex, and visual association cortex (Brodmann areas 17, 18, and 19) (Figure 4).

**FIGURE 5:**
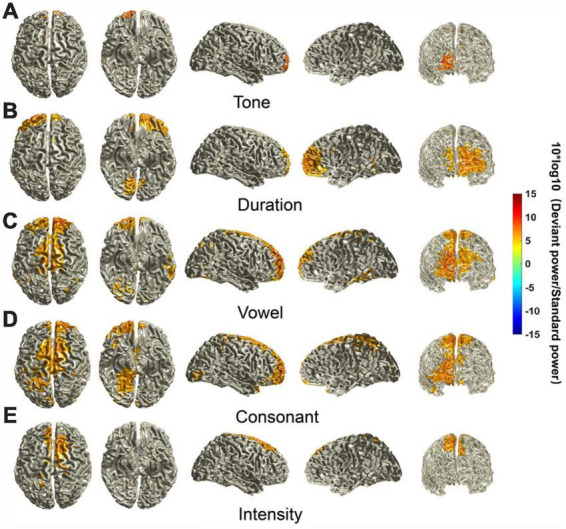
Source localization of P3a components. The location of P3a sources was defined as those with a 10 × log10 (deviant stimuli power / standard stimuli power) value in the top 40%. The mean sources of the P3a components were calculated using the ±20 ms time window around the identified time point of the peak for each different deviant.

We also analyzed the P3a-activated cortical areas. First, activated cortical areas for different deviant stimuli were all mainly in the frontal lobe, and there was little variation in the intensity of these sources. However, the regions of the cortex with activity elicited by different deviant stimuli were different. Second, the sources elicited by the vowel and consonant deviant seemed to have larger areas of strong activation, including the primary motor cortex, secondary motor cortex, frontal eye field, posterior lateral prefrontal cortex, frontopolar region, and frontal-orbital area (Brodmann areas 4, 6, 8, 9, 10, and 11). Third, there was no obvious laterality in the dominant regions (if anything, potentially biased to the right) for the vowel and consonant deviants, since most of the activated areas were near the longitudinal fissure. However, the sources elicited by the duration deviant, namely, the frontopolar region and frontal-orbital area (Brodmann areas 10 and 11), were biased to the left. Fourth, the sources elicited by the tone and intensity deviants seemed to have relatively small areas of activation (Figure 5).

### 3.4 Comparison of the sources of MMN and P3a components for the different deviant stimuli

**FIGURE 6:**
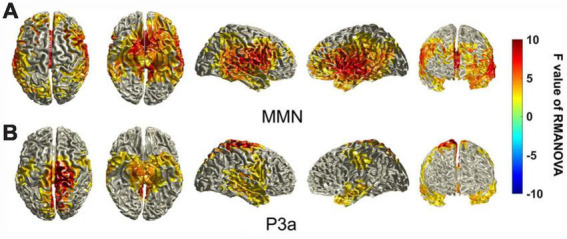
Statistically significant differences in the sources of the MMN and P3a components between different deviant stimuli. The differences in the sources of MMN/P3a components between different deviant stimuli were tested using a cluster-based random permutation procedure. A RM-ANOVA was conducted to determine the significant voxels, and the *F* values of the voxels were interpolated onto the cerebral cortex in this figure. Uncolored cortical regions represent regions that do not show significant differences (α = 5%) between different stimuli or that did not pass the cluster-based random permutation statistical correction (α = 5%, 5,000 substitutions).

For the MMN components, cortical areas that showed significantly different activation across the different deviant stimuli were mainly in the frontal lobe, temporal lobe and part of the parietal lobe. The regions showing significant differences in cortical activation were not symmetrical between the left and right hemispheres, and the frontopolar region and frontal-orbital (Brodmann areas 10 and 11) area showed activation differences only on the left side. The regions with the highest *F*-values seemed to be concentrated in the superior temporal gyrus, angular gyrus, supramarginal gyrus, auditory cortex, pars opercularis, and pars triangularis (Brodmann areas 22 and 39–45) (Figure 6A).

For the P3a components, the cortical areas that showed significantly different activation across different stimuli were different from those of the MMN components. The main difference is that the regions with the highest *F*-values seemed to be concentrated in the primary somatosensory cortex, primary motor cortex, somatosensory association cortex, secondary motor cortex, and somatosensory association cortex (Brodmann areas 1–7). These regions near the longitudinal fissure seemed biased to the right hemisphere (Figure 6B).

## 4 Discussion

Our study showed that the waveform and source location characteristics of MMN and P3a components evoked by different phonemic changes in speech sound were distinct. The vowel and consonant deviants elicited distinct MMN and P3a components, but the tone and duration deviants did not. The intensity deviant elicited distinct MMN components but did not elicit P3a components. The amplitudes of MMN and P3a components were significantly different among the deviant stimuli. The latencies of P3a components were significantly different among the deviant stimuli, but the latencies of MMN components were not. For MMN and P3a components, the activated cortical areas were mainly in the frontal-temporal lobe. However, the regions and intensities of the activated cortical areas were significantly different among the deviant stimuli. The activated cortical areas elicited by the vowel and consonant deviants seemed to have larger areas of strong activation both in MMN and P3a components compared with those elicited by tone, duration, and intensity deviants.

By using the Mandarin pronunciation multifeature paradigm to integrate the investigation of multiple phonemic changes into one sequence, this pioneering study reveals the difference in the central auditory processing of different Mandarin pronunciation elements. We did not expect that only the vowel and consonant deviants would elicit distinct MMN and P3a components. This result was different from studies based on English and Finnish pronunciation multifeature paradigms (Pakarinen et al., 2009; Sorokin et al., 2010). However, the stimuli used in these studies were non-Chinese and varied in parameter settings, making the ERP features obtained from these studies difficult to use as a reference for the results of our study. Since the subjects in our study were all normal-hearing people and all of them self-reported that they could easily distinguish the phonemic changes after the test, we ruled out the possibility that the experimental design was flawed. The nature of ERP generation is the mapping of neuronal postsynaptic potential cluster firing on the scalp. Therefore, a possible reason is that vowels and consonants carry more information for speech comprehension, and perhaps more neurons in the cortex are involved in their recognition and cognitive processing. Meanwhile, tone and duration may have less importance in speech comprehension than vowels and consonants, so fewer cortical neurons are needed. Because of the weak neural discharge and the attenuation of tissues, the obtained waveforms are not obvious (Avitan et al., 2009). Another interesting result is that intensity deviants elicited distinct MMN components but not P3a components. The MMN reflects the automatic – or semiautomatic – detection of a change in the acoustic environment (Pulvermüller and Shtyrov, 2006). However, the P3a component reflects evaluative discrimination related to the activation of an attentional switch mechanism, possibly reflecting a higher level of auditory processing (Friedman et al., 2001; Horvath et al., 2008). This is in contrast to the preattentive detection of deviant events reflected by the MMN (Friedman et al., 2001). Therefore, we deduced that although the intensity deviant could trigger the preattentive detection processing, this information may not reach the second processing stage to trigger involuntary attention switching.

Unsurprisingly, the amplitudes of MMN and P3a components showed significant differences among the deviant stimuli. The amplitude of the tone and duration MMN components were lower than that of the other three. The amplitudes of the tone, duration, and intensity P3a components were lower than the vowel and consonant P3a amplitudes. The latencies of P3a components among the deviant stimuli were significantly different, but the latencies of the MMN components were not. In fact, from the scatter diagram, we found that the latencies of the MMN components were very discrete, and similar characteristics were also observed in the P3a components for tone and duration. The latency of the ERP components reflects the difficulty of processing this acoustic information in the auditory cortex. Numerous studies have demonstrated the theoretical basis that there are differences in the processing difficulty of various elements of language in the auditory cortex (Kiefer et al., 1996; Pakarinen et al., 2007). For example, the Finnish-based study found that the latency of the vowel response was the shortest and the latency of the intensity response was the longest, but the study also found that this result was not completely consistent under different proportions of deviant stimuli (Kiefer et al., 1996). Our data did not show a similar phenomenon due to the heterogeneity of the latencies of MMN and P3a components among individuals.

Another highlight of this study is that we inferred the location of the activated sources from the potential information mapped to the scalp surface. We found that the activated cortical areas of the MMN and P3a components were mainly in the frontal lobe and included parts of the temporal and parietal lobes. This is consistent with the available data that the MMN component is generated in the frontotemporal cortex (Rinne et al., 2000; Näätänen, 2001) and the P3a component is distributed across frontal, parietal, and temporal cortical regions (Friedman et al., 2001; Wronka et al., 2012). Additionally, the regions and intensities of the activated cortical areas were significantly different among the deviant stimuli. The activated cortical areas elicited by the vowel and consonant deviants seemed to have larger areas of strong activation for both the MMN and P3a components. There was a region of strong activation in the sources of the MMN component elicited by the vowel deviant; this region was in the angular convolution and supramarginal gyrus (Brodmann areas 39 and 40) in the left hemisphere. The supramarginal gyrus is involved in phonological processing, especially phoneme discrimination and categorization, and this region is activated to enhance phonological processing and to correctly classify phonemes (Gow, 2012). Some scholars believe that phonological processes reflected by the MMN have been spatially localized to the frontotemporal cortex, with laterality to the left dominant hemisphere, emphasizing the left-hemispheric early locus of phonological processing (Pulvermüller and Shtyrov, 1991, 2006). Similar characteristics of left hemisphere dominance were also observed in the sources of the MMN components elicited by the consonant deviant. These results suggest that vowels and consonants carry more information for speech comprehension, and perhaps there are more cortical neurons that are involved in their recognition and cognitive processing. Another interesting finding is that there were large activated cortical areas in the left frontal lobe among the sources of the MMN components elicited by the intensity. However, the activated cortical areas among the sources of the P3a components were small, which generally agrees with the time domain analysis results mentioned above. Previous studies have demonstrated that the frontal cortex performs attention shifting, suggesting that the frontal cortex inhibits downstream attention or response mechanisms so that small deviations do not elicit further processing (Escera et al., 2000; Rinne et al., 2005). Therefore, we posit that the intensity stimulus may have been filtered out. Furthermore, weak or absent activated cortical areas related to the P3a components elicited by the tone and duration deviants were also observed in the superior frontal gyrus and precentral gyrus. Therefore, we can deduce that the supra-segment phonemic stimuli, e.g., the tone, duration and intensity, may require fewer higher-level auditory cognitive resources for processing.

As mentioned above, we already knew the regions and intensities of the activated cortical areas by the MMN and P3a components for each of the deviant stimuli. We further conducted a cluster-based random permutation test to find the significantly different cortical regions among the sources of the MMN and P3a components between different stimuli. As shown in Figure 6, we found that the distribution of the significantly different cortical regions among the sources of the MMN and P3a components between different stimuli is similar to the sources of MMN and P3a components shown in Figures 4, 5, which indicated that although the original source of MMN and P3a activation is mainly localized in the frontal-temporal cortex, the distribution of the active cortical regions can change dramatically (Jääskeläinen et al., 2004; Pulvermüller et al., 2005). Source localization performed on the MMN and P3a components revealed a range of underlying cortical generator clusters. The distributions of these source clusters can explain that there are numerous memory networks with different cortical regions (Pulvermüller and Shtyrov, 2006). The frontal-temporal cortex may play the main role in linguistic processes, thus allowing us to find similar cortical activation regions for different stimuli. However, based on our results, we can deduce that different Mandarin pronunciation elements elicited differential source clusters in the frontal-temporal cortex that could be linked to different types of auditory perception and cognitive processes.

There are still some shortcomings of our study. First, there is a large heterogeneity in the waveform characteristics of the MMN and P3a components among subjects, although their demographics were similar, and all of the EEG data were processed by a single data analyst. From the scatter plot in Figure 3, we found that the latencies and amplitudes of the MMN/P3a components were very discrete. Perhaps continuing to expand the number of subjects could alleviate the problem of individual heterogeneity. Second, although most of the source localization analysis results were easy to understand and explain, there were some results that were difficult to interpret. For example, as a suprasegment phonemic stimulus, the sources of the MMN components elicited by the intensity were biased to the left. However, according to a previous theory, the left hemisphere of the brain is mainly responsible for processing the linguistic information about the auditory stimulus, while the right hemisphere is mainly responsible for processing the supra-segmental features of the stimulus, such as the intensity, length, and frequency (Zatorre et al., 2002; Gandour, 2006; Pulvermüller and Shtyrov, 2006). Our results were opposite to those of existing studies. Perhaps deviant stimuli with the change of intensity also involved processing linguistic information, and the brain may process the same linguistic information as the standard stimuli. Even more difficult to understand is that the sources elicited by the intensity deviant have additional areas of activation in the occipital lobe. However, this phenomenon is often observed in hearing-impaired patients (Ortmann et al., 2017). The source localization of EEG is the reverse problem of EEG. Based on the potential signals recorded from the scalp, the position, direction and intensity information of the source of neural activity in the brain are back-calculated. Multiple factors may influence this process, and it was difficult for us to explain the unexplained cortical regions of abnormal activation.

By using high-density EEG equipment and a Mandarin pronunciation multifeature paradigm, we explored the characteristics of the auditory cortical processing procedure in response to phonemic changes in speech sounds. Based on the results of time-domain and source localization analysis, we deduced that the auditory processing centers of the brain use different auditory-related cognitive resources when processing different Mandarin pronunciation elements. Vowels and consonants carry more information for speech comprehension, and perhaps more neurons in the cortex are involved in their recognition and cognitive processing. However, suprasegment information, e.g., the tone, duration, and intensity, may not require higher-level auditory cognitive resources. Furthermore, different Mandarin pronunciation elements elicited differential source clusters in the frontal-temporal cortex that could be linked to different types of auditory perception and cognitive processing.

## Data availability statement

The original contributions presented in this study are included in this article/supplementary material, further inquiries can be directed to the corresponding author.

## Ethics statement

The studies involving humans were approved by the Medical Ethics Committee of Tianjin First Central Hospital. The studies were conducted in accordance with the local legislation and institutional requirements. The participants provided their written informed consent to participate in this study.

## Author contributions

XM: Writing – original draft. ZZ: Writing – review & editing, Investigation. YY: Investigation, Conceptualization, Writing – review & editing. YC: Data curation, Investigation, Writing – original draft. YW: Methodology, Writing – review & editing. WW: Writing – review & editing.
